# Time-resolved analysis of DNA-protein interactions in living cells by UV laser pulses

**DOI:** 10.1038/s41598-017-12010-5

**Published:** 2017-09-15

**Authors:** Angela Nebbioso, Rosaria Benedetti, Mariarosaria Conte, Vincenzo Carafa, Floriana De Bellis, Jani Shaik, Filomena Matarese, Bartolomeo Della Ventura, Felice Gesuele, Raffaele Velotta, Joost H. A. Martens, Hendrik G. Stunnenberg, Carlo Altucci, Lucia Altucci

**Affiliations:** 1 Dipartimento di Biochimica, Biofisica e Patologia Generale, Università degli Studi della Campania “L. Vanvitelli”, Vico L. De Crecchio 7, 80138 Napoli, IT Italy; 2 IRCCS SDN, Via E. Gianturco, 113, 80143 Napoli, IT Italy; 3 Department of Molecular Biology, NCMLS, Radboud University, 6500 Nijmegen, NL Netherlands; 4 Dipartimento di Fisica, Università di Napoli Federico II, Via Cinthia, 80100 Napoli, IT Italy

## Abstract

Interactions between DNA and proteins are mainly studied through chemical procedures involving bi-functional reagents, mostly formaldehyde. Chromatin immunoprecipitation is used to identify the binding between transcription factors (TFs) and chromatin, and to evaluate the occurrence and impact of histone/DNA modifications. The current bottleneck in probing DNA-protein interactions using these approaches is caused by the fact that chemical crosslinkers do not discriminate direct and indirect bindings or short-lived chromatin occupancy. Here, we describe a novel application of UV laser-induced (L-) crosslinking and demonstrate that a combination of chemical and L-crosslinking is able to distinguish between direct and indirect DNA-protein interactions in a small number of living cells. The spatial and temporal dynamics of TF bindings to chromatin and their role in gene expression regulation may thus be assessed. The combination of chemical and L-crosslinking offers an exciting and unprecedented tool for biomedical applications.

## Introduction

Dynamic binding of transcription factors (TFs) to their DNA recognition sites is crucial to mediate induction or repression of specific genes^1,2^. Interaction of DNA with proteins is a pivotal event governing cellular functions, such as transcriptional regulation, chromosome maintenance, replication and DNA repair^3^, and is critical in development and environmental adaptation. Aberrant interactions can therefore contribute to the initiation and/or progression of diseases, such as cancer. In the last twenty years, various *in vivo*, *in vitro* and *in silico* techniques have been developed^4^ to understand how these interactions, together with chromatin remodeling, occur in living cells, and to gain a better insight into this exciting area of research. The main method used to map DNA-protein interactions *in vivo* on an (*epi)*genome-wide scale^5,6^ is chromatin immunoprecipitation (ChIP) coupled with sequencing (ChIP-seq)^7^. This conventional technique binds proteins to DNA by crosslinking, generally using formaldehyde^8^. Formaldehyde reacts with an amino group of a protein side chain forming a Schiff base, which in turn reacts with an amino group of the DNA base forming a covalent bond. The enormous body of evidence on the regulatory mechanism(s) of gene expression supports the use of this chemical approach in inducing crosslinking^9–11^. However, formaldehyde-based crosslinking in ChIP has several limitations: i) formaldehyde is cytotoxic and modifies the native equilibrium between proteins and DNA; ii) formaldehyde has a short crosslinking spacer arm which is suitable for studying the interaction of proteins in proximity to DNA, but is not useful for studying proteins that bind DNA indirectly, for example proteins present in regulatory multi-complexes; iii) formation of the crosslinked polymers is slow, occurring within minutes (≈10–20 min) and thus preventing the identification of short-lived interactions. Consequently, the dynamics of biologically crucial DNA-protein associations would likely escape detection. Some findings indicate that a variety of other crosslinkers (including potential photo-physical linkers) may detect such interactions^12,13^. The ability of UV irradiation to stably crosslink DNA and proteins in different cells was reported for the first time in 1962^14^, suggesting the potential applicability of this technique in molecular studies. Unlike continuous light sources such as lamps, UV laser pulses are able to rapidly freeze DNA-protein interactions through irreversible photoreactions, allowing real-time investigation of the temporal and spatial binding of proteins on DNA. The validity of this technique has been demonstrated, mainly via *in vitro* experiments. However, a standardized approach using living cells has not yet been developed. Although our preliminary studies^15^ described DNA-histone L-crosslinking in cells, no evidence has so far been reported on the capability of L-crosslinking to freeze interactions between DNA and other proteins, such as short-lived interactions with TFs. Different factors, including experimental design and data analysis, influence the efficacy of L-crosslinking. In addition, UV irradiation causes significant DNA damage, which compromises the study of DNA-protein interactions *in vivo* ^15^. Many laser parameters need to be fine-tuned in order to obtain high efficacy and low DNA damage. The use of ultrashort femto-second (fs) laser pulses increases the yield of crosslinked DNA and significantly enhances L-crosslinking efficacy^12,13^.

ChIP following L-crosslinking (L-ChIP) could therefore be an attractive method to detect interactions between DNA and proteins in living cells^16^. Here, we demonstrate that UV laser pulses act as a zero-length crosslinker in living cells, and can be used to study both short-lived and dynamic associations in gene regulatory networks and epigenetic mechanism(s)^12,13^.

We also report on the potential applications of a novel custom tunable fs UV laser system and its integrated version with a microfluidic system designed to induce L-crosslinking in living human cells in a shorter space of time and with fewer cells compared to other methods. This UV laser device overcomes current limitations and induces highly efficient DNA-protein crosslinking. Furthermore, our pilot microfluidics data suggest that single-cell analysis may be an achievable goal. This would provide a greater insight into the functional states of individual cells, which often host rare events undetectable in bulk measurements, with major implications for biological research and medicine.

## Results

### Laser parameter settings

**Figure 1:**
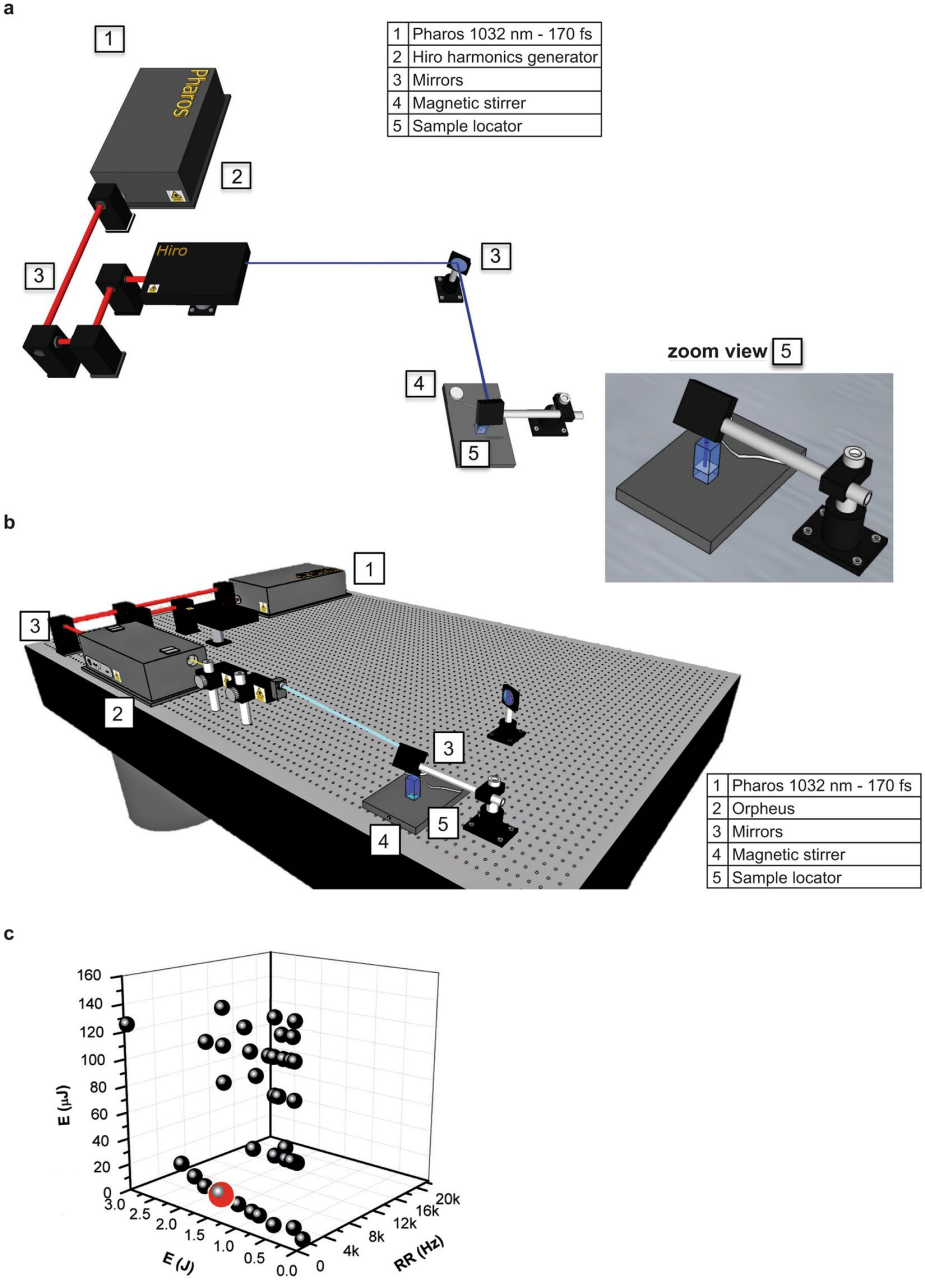
Fs PHAROS-based laser source for biological applications. Set-up of the laser system in combination with (**a**) customized harmonics generator HIRO and (**b**) optical parametric amplifier ORPHEUS. On the right, zoom view of the sample locator. (**c**) Different tested irradiation conditions in a 3D parameter space, i.e. for variable number of pulses, dose and RR used in the experimental set-up.

To optimize L-crosslinking as a method for studying DNA-protein interactions in living cells, we analyzed the crosslinking yield achieved in human cells using an advanced, flexible and user-friendly UV light source, in this case a dedicated, compact fs UV laser. Specifically, we evaluated the effectiveness of our laser system in inducing highly efficient DNA-protein crosslinking. The device, a PHAROS-based laser system (Fig. 1), is optimized to perform best in the UV domain (λ < 300 nm) at a pulse repetition rate (RR) of 2 kHz (see Materials and Methods and Supplementary Fig. S1). The high RR and high pulse and/or average energy and wavelength tunability of this laser system (Supplementary Fig. S1c) makes it an attractive tool for generating ultrashort UV pulses able to induce highly-efficient DNA-protein crosslinking in living cells. This approach is particularly useful for cell-based applications, such as ChIP (Supplementary Fig. S2), while at the same time minimizing UV light-induced cell damage.

The experiments were performed using two UV wavelengths, 258 and 300 nm, delivered by a HIRO optional wavelength converter (Fig. 1a) and an ORPHEUS optical parametric amplifier (Fig. 1b), respectively. The carrier wavelength was set at 258 nm to fit within the spectral range of 250–280 nm, in which DNA base absorption is peaked, using both continuous and ultrafast excitation^17^. Irradiation at 258 nm causes the electronic excitation of purines and pyrimidines, triggering the formation of new bonds between neighboring molecules. Specifically, UV light generates covalent binding between the reactive bases of DNA (thymine and cytosine) and amino acids (mainly phenylalanine, cysteine and tyrosine)^18–20^.

In addition, UV quanta are able to form intra- and inter-molecular thymine dimers^21^. These formations produce changes in molecular DNA structure and therefore cause disturbances in both efficiency and detection of DNA-protein crosslinking. Compared to 258 nm pulse, 300 nm wavelength strongly reduced dimer formation, leading to a much lower absorption of each base species, in particular adenine. Moreover, since the crosslinking yield at 300 nm was considerably lower than at 258 nm, this latter wavelength is preferable for L-ChIP, while 300 nm is more suitable for experiments analyzing DNA damage.

### UV laser-mediated DNA-protein interactions with low cell damage

We performed several biological experiments to assess the capability of this powerful UV light source to induce and fix DNA-protein interactions, and to establish the optimum crosslinking conditions. Our preliminary goal was to keep cell damage as low as possible while efficiently inducing L-crosslinking. A schematic representation of the experimental set-up is shown in the zoom view in Fig. 1a. A suspension of cells was irradiated with a fourth harmonic (258 nm) of the PHAROS laser source by varying energy per single pulse (E_pulse_) and RR laser parameters. The results were evaluated for L-ChIP yield, DNA-induced damage and other biological effects induced in cells by UV laser irradiation for a variable number of pulses, variable total irradiation energy (dose), and variable RR (Fig. 1c; Supplementary Fig. S1; Supplementary Tables S1 and S2). In our experimental condition, the target area illuminated by laser light was about 9–10 mm^2^. The 258 nm laser beam was collimated on the target; we thus assumed the interaction volume to be a cylinder with 10 mm^2^ base area and 5–10 mm height, depending on the overall sample amount. This results in an interaction volume of 50–100 mm^3^, i.e. 0.05–0.1 mL.

**Figure 2:**
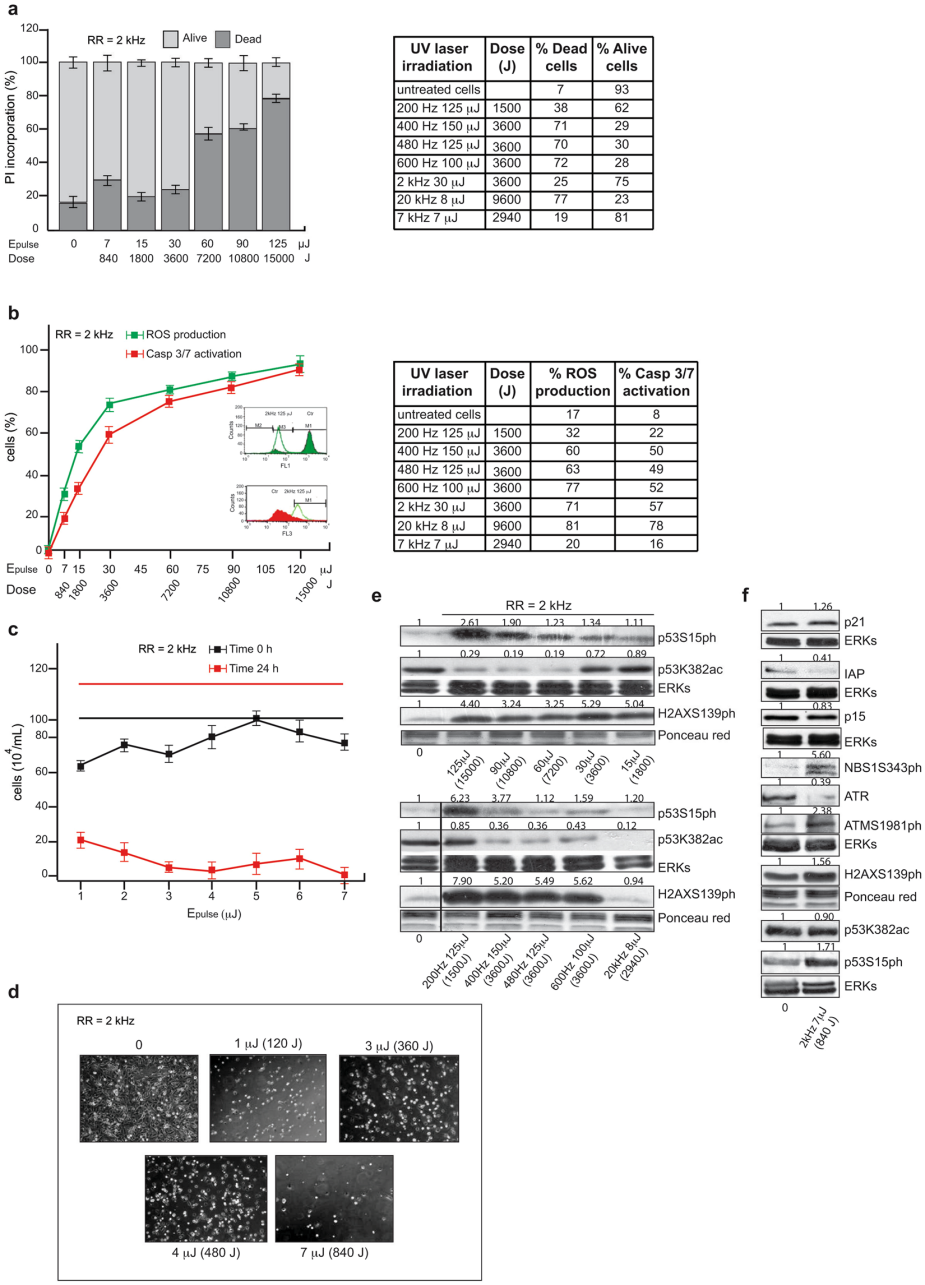
Analysis of UV-mediated cell damage. (**a**,**b**) FACS analysis in MCF7 cells irradiated as indicated: (**a**) cell death for RR at 2 kHz (left) and variable (right); (**b**) caspase-3/7 activation and ROS production for RR at 2 kHz (left) and variable (right). Values are the average of experiments in triplicate with error bars indicating standard deviation. (**c**) Cell viability analysis with trypan blue at indicated times and energies. Values are the average of experiments in triplicate with error bars indicating standard deviation. (**d**) EVOS microscope digital photographs of cells irradiated as indicated. (**e**,**f**) Western blotting analysis for indicated damage targets in MCF7 cells irradiated at RR = 2 kHz with (**d**) variable E_pulse_ and (**e**) at E_pulse_ = 7 μJ. ERKs and Ponceau red were used as loading controls. Band quantification was performed using Image J software.

MCF7 breast cancer cells (10^6^/mL) were suspended in PBS in a cuvette under magnetic stirring and irradiated (Fig. 1, zoom view). We first evaluated laser-induced cell death. Immediately after 1 min of UV exposure, cell death analysis was performed by fluorescence active cell sorting (FACS) (Fig. 2a). Notably, the percentage of dead cells measured as cells positive to propidium iodide (PI) was dependent on both energy dose and RR. For RR fixed at 2 kHz (Fig. 2a), the dose was modified by changing E_pulse_. The results show that although higher doses generally increased the percentage of dead cells, the response was not linear. After background subtraction, cell death was in fact only ≈10–20% for lower doses within 3600 J, whereas it increased to ≈50% in the 7200–10800 J range (Fig. 2a) and further increased to ≈70% at 15000 J. This observation was confirmed when the analysis was performed by varying RR and the other parameters (Fig. 2a, right table). When changing both RR and E_pulse_ and keeping the dose of 3600 J constant, we observed two distinct regimes for induced cell death: (i) at the relatively low value of E_pulse_ = 30 μJ, cell death was approximately 20%; (ii) at high values of E_pulse_, in the 100–150 μJ range, cell death increased to about 70%, regardless of the value of E_pulse_ and RR (Fig. 2a, right table). This nonlinear response of cell death induced by ultrashort 258 nm laser pulses confirmed the observations of our previous study where high values of E_pulse_ were used^15^. Taken together, our data indicate that when using a dose of significantly lower E_pulse_ values, the impact of UV pulses on living human cells is milder. This low-energy regime seems to be a good candidate for the L-ChIP technique.

To gain a mechanistic insight into cell death induced by UV laser using the two regimes, caspase-3/7 activation and reactive oxygen species (ROS) production were analyzed by FACS (Fig. 2b). UV irradiation-induced caspase-3/7 activity and ROS production were studied in a dose-dependent manner. Noteworthy, when the dose was 3600 J, caspase-3/7 activation and ROS production were ≈40% and 50%, respectively, after a background subtraction of ≈10% for caspase-3/7 and ≈20% for ROS (see untreated cell line in Fig. 2b, left table), suggesting that these processes are independent of RR. Interestingly, levels of caspase-3/7 activation and ROS production were also independent of E_pulse_, showing very similar values for both low- and high-energy UV pulses. This behavior is different from the pattern of induced cell death where two distinct regimes were used. Hence, our experimental findings suggest that at a given fixed dose, high-energy UV pulses (E_pulse_ ≥ 100 μJ) immediately kill human cells, likely due to ablation of the cell membrane, while lower energy pulses keep cells alive, triggering caspase-3/7 activation and ROS production. In agreement, cells irradiated by low energy pulses (E_pulse_ ≤ 7 μJ) at RR = 2 kHz are still alive after irradiation, but dead 24 hours later, likely due to mechanisms induced by the triggered caspase-3/7 activation and ROS production (Fig. 2c,d). Based on the results reported in Fig. 2a, we can estimate a threshold value of radiant exposure, Φ_*Th*_ = *dose*/*A*, for cell membrane ablation induced by 258 nm fs pulses, where *A* is the irradiated area, in cm^2^, of the sample cuvette. By setting E_pulse_ ≈ 20 μJ, limiting the regime to low E_pulse_ values, and excluding high E_pulse_ values where cell death takes place immediately after irradiation, we obtain:

$$\begin{array}{rcl}{{\rm{\Phi }}}_{Th} & = & \frac{{\rm{dose}}}{A({{\rm{cm}}}^{2})}\\  & = & \frac{{E}_{pulse}\cdot RR\cdot ({\rm{Irrad}}.\,{\rm{Time}})}{1({{\rm{cm}}}^{2})}\\  & \approx  & \frac{20\,{\rm{\mu }}0\cdot 2\,{\rm{kHz}}\cdot 60\,\sec .}{1({{\rm{cm}}}^{2})}\\  & = & 2.4\,{\rm{J}}/{{\rm{cm}}}^{2}\end{array}$$

To the best of our knowledge, this is the first time a similar estimate has been reported, suggesting potential advances in clinical application using fs UV pulses. Typically, UV laser pulses have so far been used for clinical applications, such as tissue ablation in corneal refractive surgery. However, this type of surgery commonly uses nanoseconds (ns) pulses, much longer than those utilized in our experiments, which focus on a completely different target system (cancer cells). For instance, a Φ_*Th*_ value of 0.21 J/cm^2^ (about 10 times lower than our estimate) is reported for 20 ns 248 nm pulses (10^5^ times longer than those used in our experiments) emitted by an excimer laser source^22–24^. Another possible comparison is with dielectric inorganic materials such as fused silica and ordinary BK7 glasses where a threshold of 1–1.5 J/cm^2^ is reported for fs laser pulses, although in the visible and not in the UV range^25^.

Taken together, these data suggest that our UV laser device is able to induce mechanism(s) of cell death associated with mitochondrial damage and caspase activation.

To further characterize the molecular pathway of damage induced by UV irradiation, we investigated protein levels of p53 phosphorylated at Serine 15 (p53S15ph)^26^ (Fig. 2e). Irradiation induced p53S15ph in a dose-dependent manner, and a robust down-regulation of its acetylated form at lysine 382 (p53K382ac). Under all conditions, the UV laser was also able to induce phosphorylation of H2AX in S139 (H2AXS139ph), a marker of double-stranded breaks (DSBs) in DNA^27^. These data are consistent with the crucial role of ATM as a mediator of cell response to DNA damage by UV exposure. The kinase activity of ATM is significantly induced upon UV damage, leading to phosphorylation and activation of NBS1, H2AX and p53, which are involved in DNA repair mechanism(s) (Fig. 2f).

**Figure 3:**
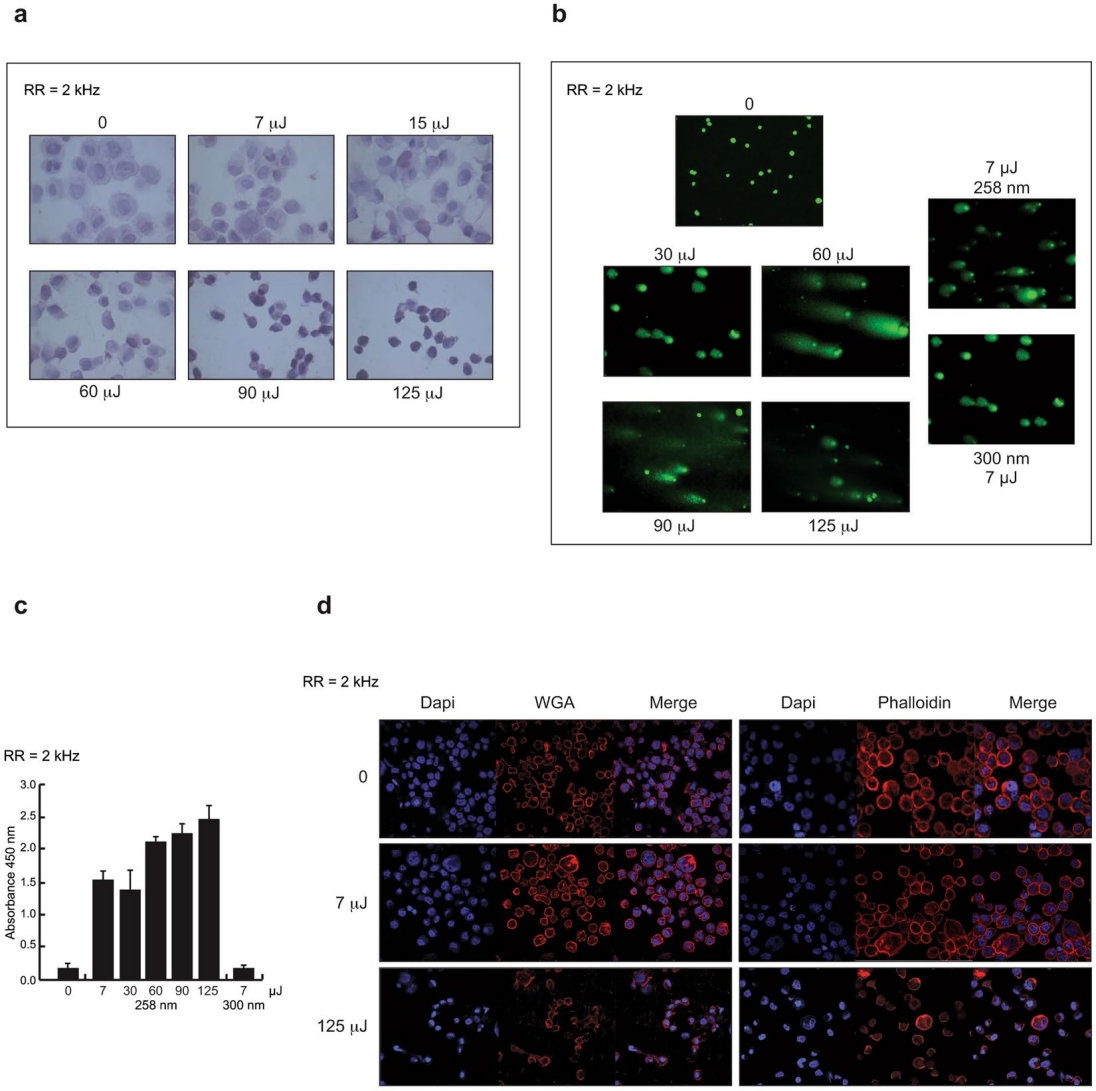
Morphological changes induced by UV laser irradiation. (**a**) H&E staining of cells irradiated at different E_pulse_ delivered at RR = 2 kHz. (**b**) Comet assay images showing intensity of DNA content in tails of cells irradiated as indicated. (**c**) Semi-quantitative measurement of CPDs in genomic DNA of cells irradiated as indicated. (**d**) Confocal images of WGA and phalloidin stainings of cells upon UV laser exposure delivered at RR = 2 kHz.

To better evaluate the morphological changes occurring upon irradiation, we performed hematoxylin and eosin (H&E) staining (Fig. 3a). The results showed that, compared to control cells, cells irradiated at E_pulse_ = 7 μJ, were intact with a round nucleus, while damage features such as nuclear condensation and cell shrinkage were clearly observed at the higher doses. To further confirm UV-induced DNA damage, we performed a comet assay and a non-isotopic immunoassay, measuring tail intensity and cyclobutane pyrimidine dimers (CPDs) in cells irradiated at different E_pulse_ delivered at RR = 2 kHz (Fig. 3b,c). The observed DNA damage was dose-dependent, reaching a maximum value at 60 μJ and a minimum value at 7 μJ. The presence of dimers at 7 μJ, albeit to a lesser extent than under other irradiation conditions, was also confirmed at a laser wavelength of 300 nm, when the presence of UV-induced pyrimidine dimers raised the background level (Fig. 3a–c). Moreover, as for 258 nm irradiation, dimer induction increased with increasing laser pulse energy, exhibiting a similar growth to that measured for ROS production and caspase-3/7 activation (Fig. 2b). Indeed, 450 nm absorbance strongly increased in the low pulse energy range to saturate at about 2.5, corresponding to ≈100% absorbance, for pulse energies higher than 120–130 μJ. This nonlinear behavior, very similar to that observed for the induction of other damage processes (ROS-caspase production/activation), suggests that bi-photonic mechanisms in the UV are most likely responsible for a large number of phenomena, including L-crosslinking.

As for the nature of the reaction mechanism at the basis of L-crosslinking, there is still a debate as to whether the step triggering photoabsorption is essentially mono-photonic or bi-photonic, as indicated in previous experiments performed *in vitro* ^12,13^ or in living cells^15^. Here, we briefly addressed this issue by changing the nature of the UV light source from an ultrashort and intense laser pulse to a continuous UV lamp, which is not able for triggering bi-photonic processes. We then attempted to carry out L-ChIP experiments using this lamp to reach an acceptable crosslinking yield. The results confirmed that this type of UV light source is not suitable for L-ChIP. The obtained process yield was always very low, if present, and the quality of irradiated samples was worse than that obtained using ultrashort laser pulses. This was due to the much greater presence of oxidized species of the bio-partner involved in the reaction. This observation corroborates the idea that a bi-photonic mechanism is at the basis of L-crosslinking, starting with a two-UV-photon absorption by a DNA base, as we previously suggested^15^.

Additionally, confocal analysis of wheat germ agglutinin (WGA) and phalloidin staining revealed morphological changes occurring in the cells upon UV laser exposure delivered at RR = 2 kHz. Compared with negative control, the cells irradiated with 7 μJ pulses exhibited a normal appearance, whereas cell morphology was significantly altered, indicating damage (Fig. 3d), when using 125 μJ pulses.

These data suggest that of all the investigated irradiation conditions, RR = 2 kHz, E_pulse_ = 7 μJ, and λ = 258 nm represent the best trade-off in terms of effective DNA-protein crosslinking induction and alteration of chromatin status.

### L-ChIP settings in living cells: defining direct binding parameters

**Figure 4:**
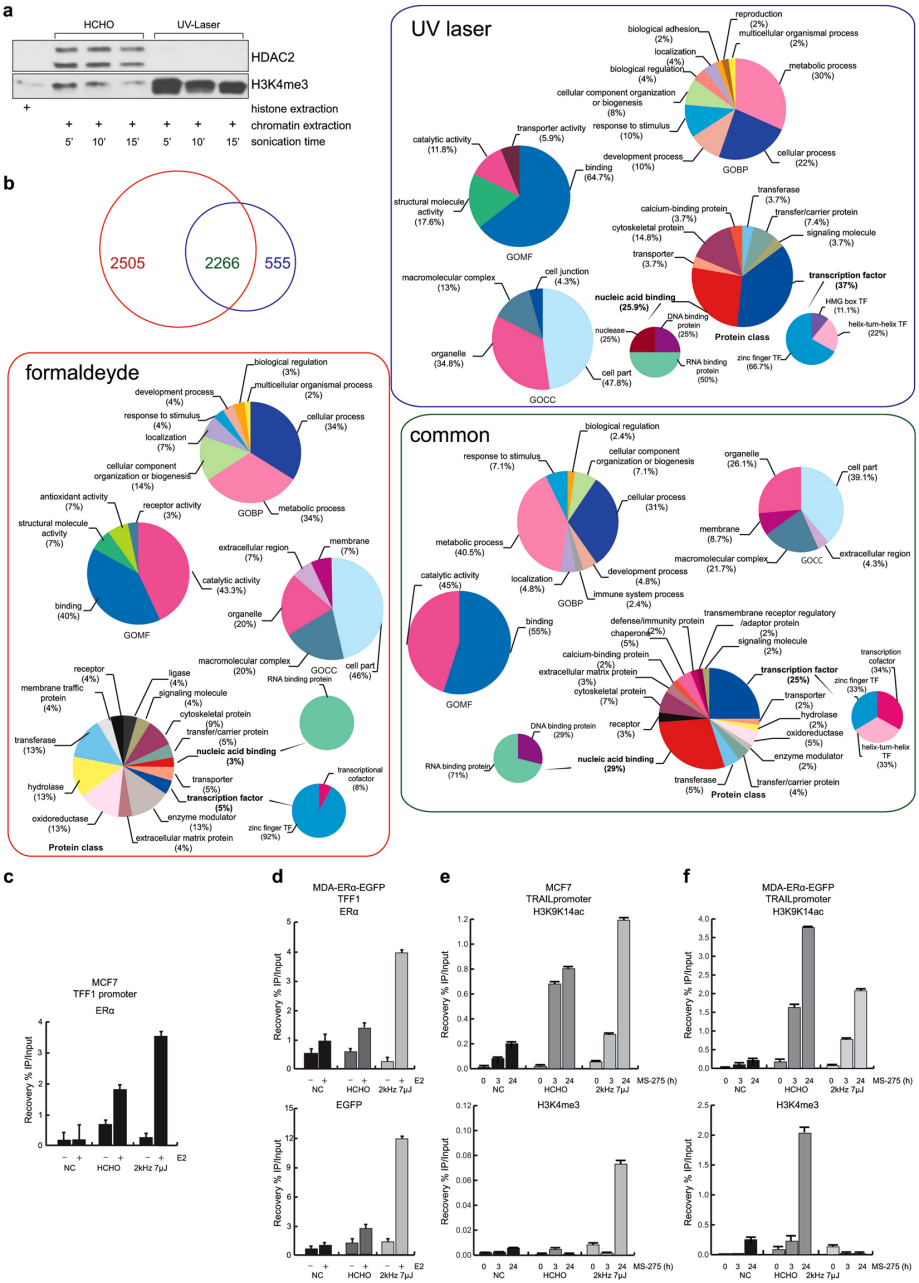
UV laser induces DNA-protein binding. (**a**) Western blotting analysis on chromatin derived from formaldehyde- and UV laser-treated cells. Histone extract was used as internal control. (**b**) Venn diagram showing number of proteins identified by MS/MS analysis within chromatin complexes derived from formaldehyde (red)- and laser (blue)-treated cells. Rectangles contain GO term analyses of each protein (formaldehyde = red, UV laser = blue and common proteins = violet). (**c** and **d**) ChIP assays performed in indicated cells treated with E2 and crosslinked with formaldehyde and UV laser showing TFF1 promoter region occupancy by (**c** and **d**, top) ERα and (**d**, bottom) GFP. (**e**,**f**) ChIP assays performed in indicated cells treated with MS-275 at indicated times and crosslinked with formaldehyde or UV laser showing TRAIL promoter region occupancy by H3K9K14ac (**e**,**f**, top) and H3K4me3 (**e** and **f**, bottom). All ChIP data obtained on immunoprecipitated fractions were normalized to input chromatin (IP/Input). Curves show the mean of at least two independent experiments with error bars indicating standard deviation.

To test the capability of the UV laser device to induce DNA-protein interactions, we performed Western blot analyses of chromatin derived from formaldehyde- or UV-treated cells. Unless otherwise specified, laser irradiation conditions were RR = 2 kHz, E_pulse_ = 7 μJ. The blots showed a higher content of histones (direct binders) and a negligible (if present) content of HDAC2 (indirect binder) in UV- compared to formaldehyde-treated samples (Fig. 4a), suggesting that L-crosslinking mainly identifies direct DNA-protein interactions in these settings (Supplementary Fig. S3a). To confirm and corroborate this hypothesis, we performed mass spectrometry analysis to identify proteins within the chromatin complexes derived from formaldehyde- and UV-treated samples (Fig. 4b; Supplementary Fig. S3b). Our analysis identified 2505 and 555 specific binder groups to formaldehyde and UV treatment, respectively, as well as 2266 common binders (Fig. 4b). In line with Western blot results, gene ontology molecular function (GOMF) term analysis by category counting of identified proteins indicated that the category “DNA binding” was significantly enriched in the UV laser-treated samples (p < E = −76) (Fig. 4b; Supplementary Table S3). Moreover, gene ontology protein class (GOPC) term analysis highlighted enrichment of the categories “transcription factor” and “nucleic acid binding” from 5% obtained in formaldehyde-treated samples to 37% and 26% in UV-laser treated samples, respectively (Fig. 4b). A more detailed analysis of these two categories showed a different identity of the classified proteins in the differently treated samples. Specifically, L-crosslinking led to a 25% enrichment of DNA-binding proteins within the “nucleic acid binding” category, compared to 100% of RNA-binding proteins obtained with formaldehyde crosslinking (Fig. 4b). Taken together, these data strongly suggest that the UV-laser device is able to fix interactions between DNA and nearby proteins, preferring direct binders to DNA.

### Detection of direct protein binding to DNA by ChIP assay

To better identify the interactions of proteins with DNA, we performed ChIP assays in living cells using formaldehyde (X-ChIP) and UV laser (L-ChIP) as crosslinker (Supplementary Fig. S2). The L-ChIP protocol used to analyze the interaction between chromatin and nuclear receptors driven by a UV laser energy source was as previously described^28^. We assessed how the laser device impacts estrogen receptor alpha (ERα) binding to promoters of estrogen (E2) target genes, such as TFF1. Although stimulation of MCF7 breast cancer cells with E2 radically increased ERα occupancy on TFF1 (Fig. 4c), L-ChIP yielded much higher detection sensitivities (about 100%) than X-ChIP. This is, to the best of our knowledge, the first report of a UV laser source that more efficiently freezes the binding of a TF to its DNA recognition site.

Since ChIP experiments are lengthy and expensive, and the space of experimental parameters potentially affecting L-ChIP yield is vast, resulting in a huge number of laser setting combinations to be tested, we devised a strategy for an efficient L-ChIP yield optimization procedure based on a fast screening preliminary step of the tested conditions. To this end, we generated MDA-MB-231 breast cancer cells, negative for ERα and stably expressing GFP-ERα fusion protein, to use as an alternative model to MCF7 cells and as a fast model to screen the optimal irradiation condition to test then in L-ChIP assay (Supplementary Fig. S4 and Materials and Methods). This fast screening step is shown in Supplementary Figure S4 and represented by the indicator “Fluorescence” (olive green line) in Supplementary Tables S1 and S2. The idea was to screen the laser parameters able to induce high values of fluorescence assumed to be an indicator of the occurrence of L-crosslinking, since the final fluorescence signal is proportional to the amount of GFP-ERα fusion protein bound to ERE sequences. Several quantitative levels of the measurements taken are reported in Supplementary Tables S1 and S2. By integrating and combining the results derived from the different experiments, the condition RR = 2 kHz, E_pulse_ = 7 μJ, and λ = 258 nm was also found to be optimal for L-ChIP. Thus, hormone-deprived cells were treated with 10 nM E2 for 1 hour, and crosslinked DNA-bound ERα complexes were subsequently isolated by ChIP using anti-ERα or anti-GFP. Noteworthy, L-ChIP led to a 3- and 4-fold enrichment of ERα and GFP binding, respectively, upon E2 stimulation at the TFF1 promoter compared to X-ChIP (Fig. 4d)^29^. Notably, treatment times for X-ChIP and L-ChIP were 10 min and 1 min, respectively, indicating that L-ChIP is more suitable for studying short-lived interactions.

In line with these findings, the fluorescence intensity of TFF1-ERα complexes extracted from ERα-GFP-MDA-MB-231 cells during L-ChIP and X-ChIP showed higher signals after laser treatment (Supplementary Fig. S4 and Materials and Methods). Thus, we show that our UV laser source enhances crosslinking, above all in terms of direct DNA-protein binding.

To further strengthen the hypothesis that L-crosslinking preferentially detects direct DNA-protein binding, we investigated histone mark modifications triggered by specific drugs at well-known chromatin regions^30^. Specifically, we analyzed the enrichment of histone H3 acetylated at lysines 9 and 14 (H3K9K14ac), and trimethylated at lysine 4 (H3K4me3) upon treatment of breast carcinoma cell lines with MS-275, a well-known histone deacetylase (HDAC) inhibitor able to induce chromatin hyperacetylation (Fig. 4e,f)^31^. Based on our previous findings^30,32^, expression of the death ligand TRAIL is up-regulated in cells treated with MS-275 through an increase in H3K9K14ac and H3K4me3 on its promoter region. In order to study the dynamics regulating TRAIL expression, we used L-ChIP to investigate levels of acetylated and trimethylated histone H3, using X-ChIP as control. As shown in Fig. 4e and f, the TRAIL promoter region in MCF7 and MDA-MB231 cells (as well as in hematological cancer cells, such as U937 and NB4 cells, data not shown) is mainly hyperacetylated, but also trimethylated, in a time-dependent manner upon 3 and 24 hours of treatment with 5 μM MS-275. Higher levels of promoter-associated H3K4me3 were observed in all MS-275-treated cells compared with untreated parental cells. No signal was obtained from non-irradiated immunoprecipitated samples (Fig. 4e,f).

Taken together, these data suggest that L-ChIP may be a useful method to detect direct and transient binders to DNA in living cells.

### Scaling down to small cell samples by coupling a microfluidic system to UV laser

In order to potentiate L-crosslinking as a method for studying DNA-protein interactions in smaller cell samples, we analyzed the effects and degree of crosslinking achieved by using the fs UV source combined with a customized microfluidic cell system (Sigolis, http://hornonline.com/sigolis-ab/).

**Figure 5:**
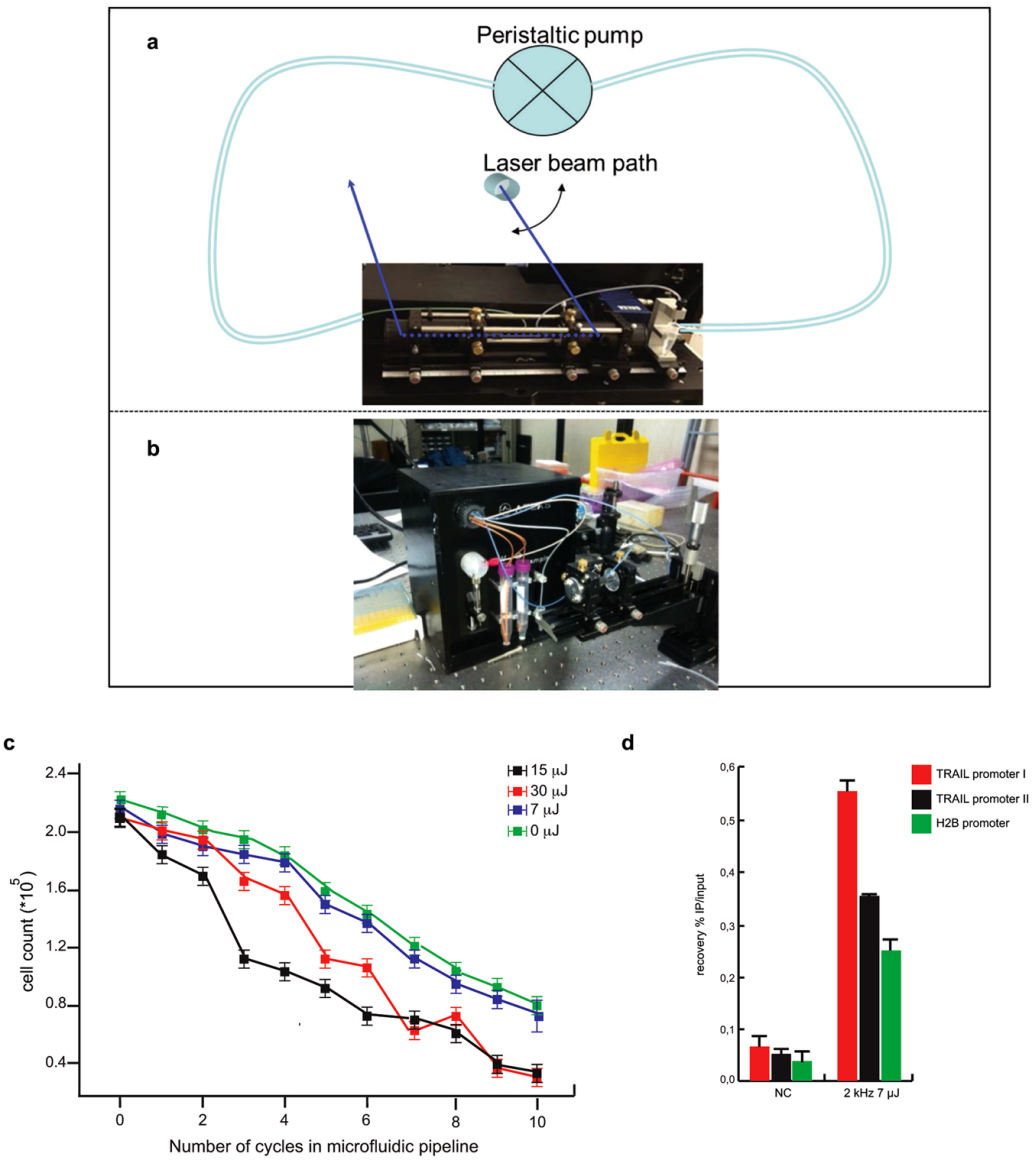
Potential use of a custom microfluidic device in L-ChIP. (**a**) First and (**b**) second set-up of microfluidic system used. (**c**) Proliferation curve by trypan blue of MDA-ERα-GFP irradiated at the indicate energy doses. (**d**) ChIP assays performed in cells irradiated at E_pulse_ = 7 μJ and RR = 2 kHz for 8 passes showing TRAIL promoter region occupancy by H3K4me3. ChIP data obtained on immunoprecipitated fractions were normalized to input chromatin (IP/Input). Curves show the mean of at least two independent experiments with error bars indicating standard deviation.

Our first experimental set-up (Fig. 5a) allowed irradiation of the target solution, while stirred, in a cuvette. However, this arrangement presented two practical drawbacks: i) beam intensity was inversely proportional to the thickness of medium travelled, and ii) interaction volume was dependent on the distance from beam focus (see Materials and Methods). To better control irradiation conditions, we switched to a second irradiation set-up (Fig. 5b), where the target solution was circulated in a prototype microfluidic device built in-house. Here, the sample was pumped into appropriate bio-compatible and flexible Tygon tubes by a peristaltic pump. This set-up can be operated either as a closed fluidic circuit, or in multipass irradiation configuration, where the sample is pumped back and forth and irradiated at each pass (see Materials and Methods).

Initially, several flow speed values were tested, all within a 25–150 μl/min window, to ensure a laminar flow of cell solution. The irradiation settings of our targets lasted from a few seconds to a few minutes.

We first evaluated the effects of this custom device in inducing cell death. Based on the capability of the instrument to carry out multipass irradiation, suspensions of MDA-ERα-GFP (1 ∗ 10^6^/mL) were passed (from 1 to 10 times) at a flow speed of 25 μL/min (the optimal condition chosen from a series of settings, data not shown). Analyses were performed by fixing RR = 2 kHz and varying E_pulse_ (Fig. 5c). As expected, cell death was dependent on both E_pulse_ and the number of passes inside the tube.

The irradiation condition able to induce the lowest percentage of cell death, comparable to non-irradiated cells, was RR = 2 kHz and E_pulse_ = 7 μJ, while the highest percentage of cell death was induced by RR = 2 kHz and E_pulse_ = 15 μJ (Fig. 5c). L-ChIP was therefore performed on cells irradiated at the optimal RR = 2 kHz and E_pulse_ = 7 μJ condition for 8 passes, representing a good trade-off between the highest possible crosslink yield and limited laser-induced damage in irradiated cells. Interestingly, L-ChIP provided a good recovery of H3K4me3 in different promoter regions, higher than that obtained with ordinary cuvette irradiation (Fig. 5d).

These results can be considered as a proof-of-principle, and indicate that combining L-ChIP with a microfluidic approach could facilitate the analysis of (epi)genetic networks in small cell populations. Using this combination, we obtained a factor of 10 decrease (from 10^6^ to 10^5^) in the number of crosslinked cells contained in the sample, compared to L-ChIP alone. We can therefore envisage a noticeable improvement in terms of studying smaller and smaller cell samples. Further optimizing the architecture of the microfluidic device as well as miniaturizing flow cells and tubing might lead in the near future to a scaling down to single-cell investigation/crosslinking, thus obviating the large number of cells required by conventional ChIP.

## Discussion

All biological functions depend on finely tuned interactions of proteins with DNA that trigger specific cellular responses^33^, a key issue in all the biosciences. In the last few years, enormous efforts have been made to develop and refine a wide range of techniques to study DNA-protein interactions and to provide a spatiotemporal map of these functional interactions inside the cell.

Recent advances in *in vivo* analysis of DNA-protein complexes have been achieved by ChIP assay. Many DNA-protein interactions occur in a short space of time (even less than 1 min), as the complexes involved are easily formed and easily broken with lower dissociation constants. Transient interactions are crucial in explaining the dynamic nature of cellular responses. However, bindings activated in transient interactions are difficult to detect experimentally, hampering our understanding of the precise timing of expression and gene regulation. One major problem is that the efficacy of chemical crosslinkers, such as formaldehyde, is limited by their extended reaction times, which are much longer than those typical in transient interactions.

Here, we demonstrate the validity of L-crosslinking in living cells using ultrashort UV laser pulses to freeze the reactive DNA bases (cytosine and thymine) to amino acids of a neighbor protein. Specifically, we demonstrate for the first time that L-crosslinking acts as a zero-length method causing less damage to cell integrity at specific settings.

This approach is, in fact, able to crosslink a nuclear protein such as H3ac, known to be in close contact with DNA during its interaction, with much higher efficiencies than those obtained with formaldehyde-based chemical crosslinking. We first characterized the impact of UV laser irradiation on living cells across a range of laser parameters such as energy dose and RR, largely confirming our previous data^15^ (Fig. 2). Interestingly, at a given dose, lower values of E_pulse_ trigger the lowest cell death immediately after irradiation, suggesting that nonlinear mechanisms are responsible for this process. By designing and testing the experimental set-up shown in Fig. 1, we established that the irradiation condition E_pulse_ = 7 μJ delivered at RR = 2 kHz at λ = 258 nm was the most efficient DNA-protein L-crosslinker, causing the lowest cell damage in terms of cell death, caspase-3/7 activation and ROS production, changes in cellular morphology, and activation of damage targets (Figs 2 and 3). By Western blotting analysis of chromatin derived from formaldehyde and UV-treated samples, we obtained initial evidence that our UV laser is able to fix direct binders on DNA (Fig. 4a). While chemical crosslinking detected both a direct DNA-protein interaction, with the H3K4me3 histone, and an indirect interaction, with HDAC2, L-crosslinking only detected the former. Crucially, this finding demonstrates that a combination of the two methods is able to distinguish direct from indirect interactions.

Mass spectrometry analysis of chromatin derived from formaldehyde- and L-crosslinked samples clearly supported our findings, providing a greater insight into the identity of chemical- and UV-induced DNA-protein complexes after crosslinking. A global screening of the crosslinked proteins revealed that our UV laser system is mainly able to link direct binders on DNA, as suggested by GOMF and GOPC analyses (Fig. 4b).

Our data further demonstrate and validate UV laser irradiation as a useful tool to detect direct as well as transient binders to DNA (Fig. 4c–f). Notably, *in vivo* ERα-, H3K9K14ac- and H3K4me3-DNA complexes are detected with unprecedented sensitivity and temporal resolution, exceeding the efficiency of formaldehyde. By combining different temporal and spatial measurement schemes, based for instance on labeled cellular models (such as MDA-ERα-GFP), this technique may allow the analysis and mapping of the dynamic assembly/disassembly of a specific protein/TF on its cognate DNA site in working cells under a variety of conditions. A better understanding of the dynamics of DNA-protein interactions could be achieved using a next generation sequencing approach, and is crucial to fully unravel a specific cellular process.

Importantly, L-crosslinking can also be adapted to different model systems and further modified, by using a labeling technique, to increase its resolution and applicability.

Finally, ChIP requires large cell samples, and reducing the number of cells needed to perform this type of assay represents a major challenge^34^. The methodology presented here overcomes this limitation and provides a proof-of-principle that L-crosslinking coupled with a suitable microfluidic device is able to induce DNA-protein crosslinking in small cell numbers (10-fold decrease) (Fig. 5).

Our preliminary microfluidics data are very promising, potentially leading the way towards single-cell analyses, crucial for our understanding of disease and the discovery of new druggable targets. Further development of the technology will help elucidate the puzzling differences between heterogeneous cell populations. Such insights will mark an important step towards a personalized approach for the prognosis, diagnosis and therapy of a particular disorder.

## Methods

### UV laser source

Our laser source, based on a PHAROS pump laser (Light Conversion; Fig. 1), was built as part of the EU funded project ATLAS (221952). The pump laser is an fs PHAROS system, whose active medium is a Yb:KGW crystal matrix delivering 180 fs pulses centered at a wavelength of 1030 nm, with E_pulse_ up to 1.5 mJ and RR tunable within single shot operation to 200 kHz with optimized performances in the UV domain around 260 nm at 2 kHz (Fig. 1a; see Supplementary Fig. S1a for PHAROS output response versus laser RR). PHAROS pumps a harmonics generator (HIRO; Light Conversion), which produces high-energy II (515 nm), III (340 nm), and IV (258 nm) harmonic pulses of the fundamental radiation (see Supplementary Fig. S1b for 515 nm and 258 nm radiation). Either alone or combined with HIRO for double pulse generation, PHAROS pumps an optical parametric amplifier (ORPHEUS; Light Conversion), tunable within a wide spectral window, between 0.2 and 2.6 μm (Fig. 1b), which has a spectral response (Supplementary Fig. S1c). The experimental set-up used in this study is shown in Supplementary Fig. S1a and b. As a further option, PHAROS can directly pump an additional HIRO harmonics generator to generate low-energy UV pulses delivered at high RR, up to 80 MHz, as in single photon counting experiments (Supplementary Fig. S1d).

### Cell lines and reagents

MDA-MB-231 and MCF7 cells (American Type Culture Collection) were tested and authenticated following the manufacturer’s instructions. Cell lines were grown in Dulbecco’s Modified Eagle’s Medium (DMEM; Euroclone) supplemented with 10% heat-inactivated fetal bovine serum (FBS; Sigma) and antibiotics (100 U/mL penicillin, 100 mg/mL streptomycin and 250 mg/mL amphotericin-B). Transfected MDA-ERα-GFP cells were grown in presence of 0.5 mg/mL G418 (Invitrogen). All cell lines were maintained in an incubator at 37 °C and 5% CO2 in a fully humidified atmosphere. MS-275 and estradiol were purchased from Alexis and Sigma, respectively.

### Cell cycle and cell death analysis

The cells, irradiated and non-irradiated, were resuspended in 500 μL of a hypotonic solution containing 1X PBS, 0.1% sodium citrate, 0.1% NP-40, 50 μg/mL PI and RNAse A. After 30 min in the dark, samples were acquired on FACSCalibur flow cytometer (BD Bioscences) using Cell Quest software (BD Biosciences) and analyzed using ModFit LT version 3 software (Verity), as previously reported^35,36^. Cell death was measured as sub-G1 DNA fragmentation. All experiments were performed in triplicate and values expressed in mean +/−SD.

### Caspase assay

Caspase-3/7 activity was detected within living cells using a B-Bridge kit with cell-permeable fluorescent substrates, following the manufacturer’s instructions. Cells were washed twice in cold PBS and incubated for 1 h in ice with the substrate FAM-DEVD-FMK. Cells were analyzed using Cell Quest software applied to FACScalibur (BD Biosciences). Experiments were performed in triplicate and values expressed in mean +/−SD.

### ROS production

To evaluate ROS production, the cells were incubated with 5 μM dihydroethidium (Invitrogen). After 30 min in the dark, the cell suspension was analyzed by FACSCalibur (BD Bioscience). Experiments were performed in triplicate and values expressed in mean +/−SD.

### Cell viability analysis

At irradiation times indicated in the Results section, the cells were incubated with trypan blue (Euroclone) at a ratio of 1:1. Trypan blue-positive cells (dead cells) as well as total cell population were counted using an optical microscope to calculate the percentage of viable cells.

### Western blotting

Western blot analyses were performed following the antibody suppliers’ recommendations. Antibodies used were: p53S15ph (R&D Systems), p53K382ac (Upstate), H2AXphS139 (Abcam), p21 (BD Biosciences), IAP (Santa Cruz Biotechnology), p15 (Santa Cruz Biotechnology), NBS1S343ph (Cell Signaling), ATR (R&D Systems), ATMS1981ph (Abcam), ERKs (Santa Cruz Biotechnology), HDAC2 (Abcam), H3K4me3 (Diagenode), ERα (Santa Cruz Biotechnology), GFP (Abcam).

### Optical microscopy and digital imaging

Cells were imaged under an EVOS phase-contrast microscope (AMG), and images were captured via integrated digital camera. Experiments were performed in triplicate.

### Hematoxylin and eosin staining

Cells were seeded (1 ∗ 10^5^ cells/mL) in triplicate on glass coverslips and fixed in fresh 4% paraformaldheyde (PHA) at room temperature for 10 min. They were then stained with H&E (Sigma) and observed under light microscope (Zeiss).

### Alkaline comet assay

Alkaline comet assay (CometAssay, Trevigen) was used to detect DNA after UV laser irradiation, following the manufacturer’s instructions and as previously reported^37^. Images were acquired using an epi-fluorescence microscope (Zeiss). Data analysis was performed with CometScore software (free version) to determine intensity of the comet tail.

### Cyclobutane pyrimidine dimer evaluation

CPDs in genomic DNA of cells after UV irradiation were detected in living cells using an Abnova kit, following the manufacturer’s instructions. Cells after irradiation were fixed/denatured and then incubated for 1 h at room temperature with anti-CPD antibody and then with HRP-conjugated secondary antibody. After incubation with substrate reagent, the reaction was stopped and absorbance was measured using an Infinite M200 (Pro) (Tecan) multimode reader at 450 nm. Experiments were performed in triplicate and values expressed as mean +/−SD.

### Phalloidin staining

Actin filaments were stained with Alexa Fluor 647 phalloidin (Invitrogen). Briefly, cells were fixed onto glass coverslips with fresh 4% PHA for 10 min, permeabilized in PBS containing 1% Triton X-100 (PBST) for 5 min, and blocked in 1% bovine serum albumin for 30 min; 647 Phalloidin was then added for 30 min at 0.5 μg/mL as well as DAPI staining for nuclei (0.1 μg/mL DAPI in PBS for 1 min) in the dark. Slides were mounted with ProLong Antifade reagent (Invitrogen) and visualized under confocal microscope (red filter; Zeiss 700).

### WGA staining

Sialic acid residues were stained with Alexa Fluor 647 WGA (Invitrogen) following the manufacturer’s instructions. Briefly, cells were fixed onto glass coverslips with fresh 4% PHA for 15 min and then stained with WGA for 10 min at 5 μg/mL. Slides were mounted with Prolong Antifade reagent (Invitrogen), and visualized under confocal microscope (red filter; Zeiss 700).

### Mass Spectrometry

In preparation for mass spectrometry, crosslinked proteins were lysed to obtain nuclei, washed with urea and sonicated for 10 cycles at max power (50% duty cycles). Obtained chromatin was dialyzed and treated with DNAseI (10 U for 10 min at 37 °C). Subsequently, proteins were reduced with 0.1 M dithiothreitol and denatured using 8 M urea. Proteins were alkylated using iodoacetamide and digested overnight with trypsin (enzyme to protein ratio, 1:100). Obtained peptides were acidified with trifluoroacetic acid and desalted using stage tip protocol. Peptides were separated using NanoLC (Sciex) connected online to a mass spectrometer (LTQ-Orbitrap; Thermo Fisher Scientific). Raw data were analyzed using MaxQuant 1.3.0.5 software^38^. Identical proteins were filtered for known contaminants and reverse hits, as well as hits without unique peptides.

### Chromatin immunoprecipitation

ChIP experiments were performed as previously described^28,32^. Antibodies used were: ERα (Santa Cruz Biotechnology), GFP (Abcam), H3K9K14ac (Diagenode), H3K4me3 (Diagenode). Primer sequences were as follows: PS2/TFF1 promoter region (at −393 and −71 from +1), forward (5′-ggc cat ctc tca cta tga atc-3′) and reverse (5′-ggc agg ctc tgt ttg ctt aaa-3′); TRAIL promoter region (at −229 and −46 from +1), forward (5′-agt ttc cct cct ttc caa cg-3′) and reverse (5′-cac tga agc cct tcc ttc tct-3′).

### Establishment of MDA-ERα-GFP cell line

pERα-GFP-C1 vector (kindly provided by Ken-Ichi Matsuda, Department of Anatomy and Neurobiology, Kyoto Prefectural University of Medicine) was transfected into MDA-MB231 cells with Lipofectamine 2000 (Invitrogen), following the manufacturer’s protocol. To quantify ERα-GFP expression, cell medium was aspirated and cells were washed with PBS, and then trypsinized and resuspended in PBS. Instrument settings were calibrated using mock transfected and non-transfected cells. Cells were then analyzed using dot plots measuring forward versus side scatter as well as histogram plots measuring count values of FL-1 (green fluorescence). Using the flow cytometry values as described above, the region of interest was then selected for the cell sort gating threshold. Sorted lines were cleansed with 70% ethanol and buffered with sterile PBS. Positive-gated cells were collected in tubes that were pre-incubated in cold FBS. Tubes were centrifuged and cells were re-plated in DMEM medium. The yield of cells recovered in each sort was approximately 80% of the number of cells gated and counted by the flow cytometer. Media was replenished after approximately 4–5 h after the sort to remove any additional contaminants or debris remaining once the sorted cells adhered to the cell culture plates. For details see also Supplementary Materials and Methods.

### In-house prototype microfluidic device

In order to better control irradiation conditions, we switched from a cuvette-based irradiation scheme to a second irradiation set-up, where the target solution circulated in a prototype microfluidic device built *in-house*. Here, the sample was pumped into appropriate biocompatible and flexible tygon tubes, with an inner diameter of 0.63 mm, by a peristaltic pump. The fluidic circuit could be either closed or terminating in a cuvette, thus allowing for multipass irradiation, with the sample being pumped back and forth. Several values for the flow speed were tested, all compatible with a laminar flow of the solution, within a 25–150 μL/min window. Typically, irradiations of our targets lasted from a few seconds to a few minutes.

The fluidic circuit contained a capillary ending with two quartz windows, so as to feed the UV pulses. When the sample was irradiated by laser pulses, the capillary had an inner diameter of about 1 mm. After optimizing the device, no significant leak was measured from the fluidic connections and cell death due to mechanical stress alone, with no laser-induced cell irradiation, was reduced to ≈2% per cycle.

## Electronic supplementary material

Supplementary Information

Table S3 MS analysis
