# Supplementary material for: Time-resolved analysis of DNA-protein interactions in living cells by UV laser pulses
Source: Sci Rep. 2017 Sep 15;7:11725. doi: 10.1038/s41598-017-12010-5 (PMC5601431; doi:10.1038/s41598-017-12010-5)
Supplement: Supplementary file 1 — Supplementary Information [file 41598_2017_12010_MOESM1_ESM.pdf]

**Time-resolved analysis of DNA-protein interactions in living cells by UV laser pulses**

Angela Nebbioso[1,*,§], Rosaria Benedetti[1,§], Mariarosaria Conte[2], Vincenzo Carafa[1], Floriana De Bellis[1,3], Jani Shaik[3], Filomena Matarese[3], Bartolomeo Della Ventura[4], Felice Gesuele[4], Raffaele Velotta[4], Joost HA Martens[1,3], Hendrik G. Stunnenberg[3], Carlo Altucci[4,*], Lucia Altucci[1,*].

[1] Dipartimento di Biochimica, Biofisica e Patologia Generale, Università degli Studi della Campania "L. Vanvitelli", Vico L. De Crecchio 7, 80138, Napoli, IT

[2] IRCCS SDN, Via E. Gianturco, 113, 80143, Napoli, IT

[3] Department of Molecular Biology, NCMLS, Radboud University, 6500 Nijmegen, NL

[4] Dipartimento di Fisica, Università di Napoli Federico II, Via Cinthia, 80100, Napoli, IT

[§] Equal first authors

*To whom correspondence should be addressed. Tel: +390815667569; Fax: +39 081450169; Email: lucia.altucci@unicampania.it

Correspondence may also be addressed to Angela Nebbioso. Tel: +390815665682; Fax: +39081450169; Email: angela.nebbioso@unicampania.it and Carlo Altucci. Tel. +39081679286; Email carlo.altucci@unina.it

## Abstract

**Immunoprecipitation assay**

MDA-ERα-GFP extracts were incubated in NP-40 (0.5%), Tris-HCl pH 8.0 (20 mM), NaCl, (150 mM), PMSF (1 mM), 10% glycerol, EDTA (1 mM) and 1X protease inhibitor mix (Sigma) for 20' on ice. Cell debris was removed and the soluble incubated with anti-GFP and anti-ERα overnight at 4°C. The immune complexes were isolated by adding agarose-protein A/G Plus (Santa-Cruz) for 2h at 4°C. Proteins were then eluted, resuspended and analyzed by Western blot.

**Immunofluorescence experiment**

MDA-ERα-GFP cells were grown on gelatin-coated slides and then incubated with anti-ERα (Santa Cruz) according to manufacture's instructions. Nuclei were stained with Hoechst for 10 minutes; images were analyzed by fluorescence microscope (Zeiss).

**In Fluorescence Experiments**

Cells, irradiated and not were gently lysed to preserve the integrity of nuclear membranes. Nuclei were then sonicated and the chromatin was recovered. The protein ERα-GFP linked to DNA (on ERE sequences) was purified and the quantity of GFP signal was measured by a multimode reader Infinite M200 Pro (Tecan) at 485 nm and 520 nm.

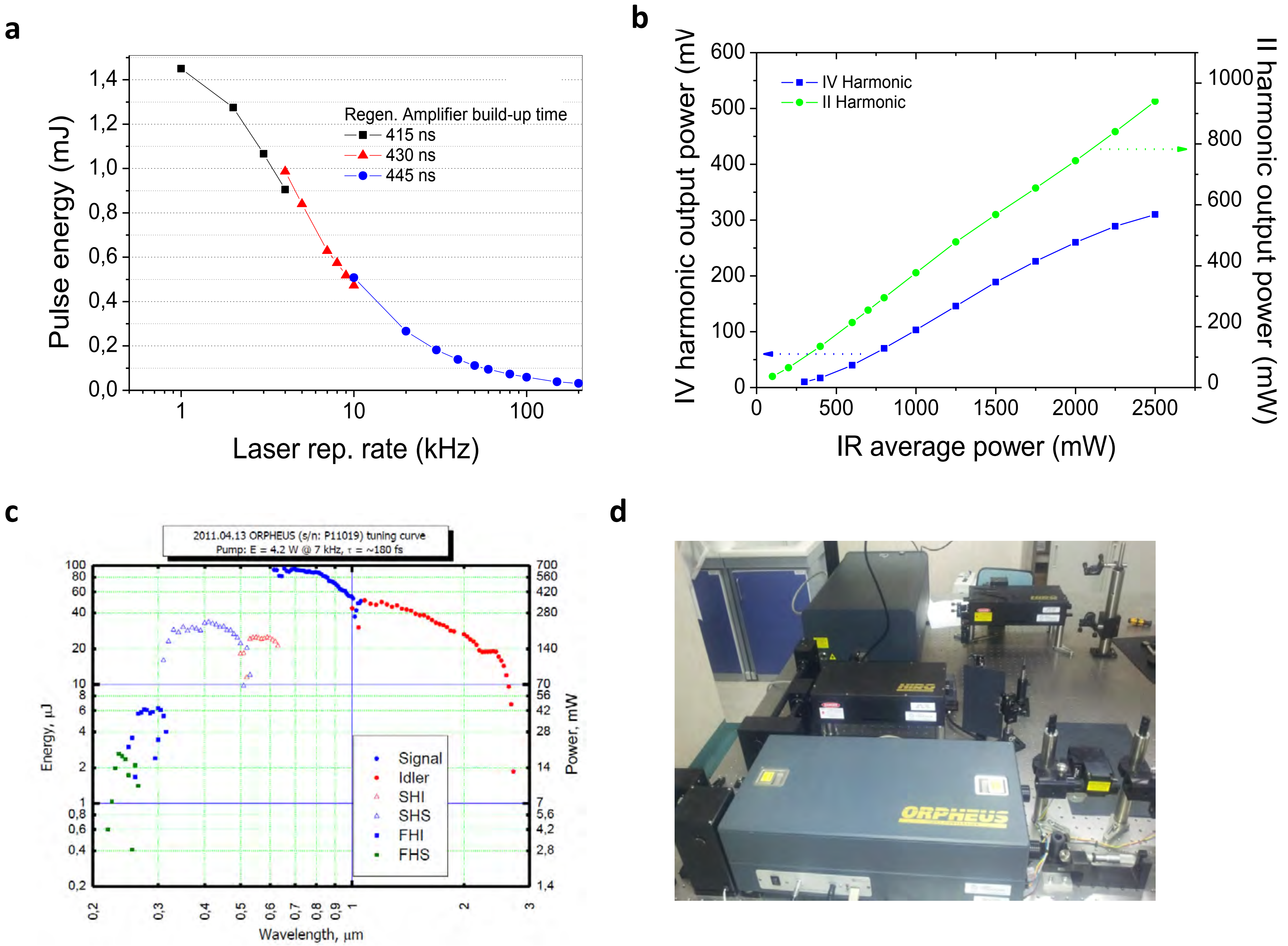


**Supplementary Figure S1: Pharos-based laser parameter settings**

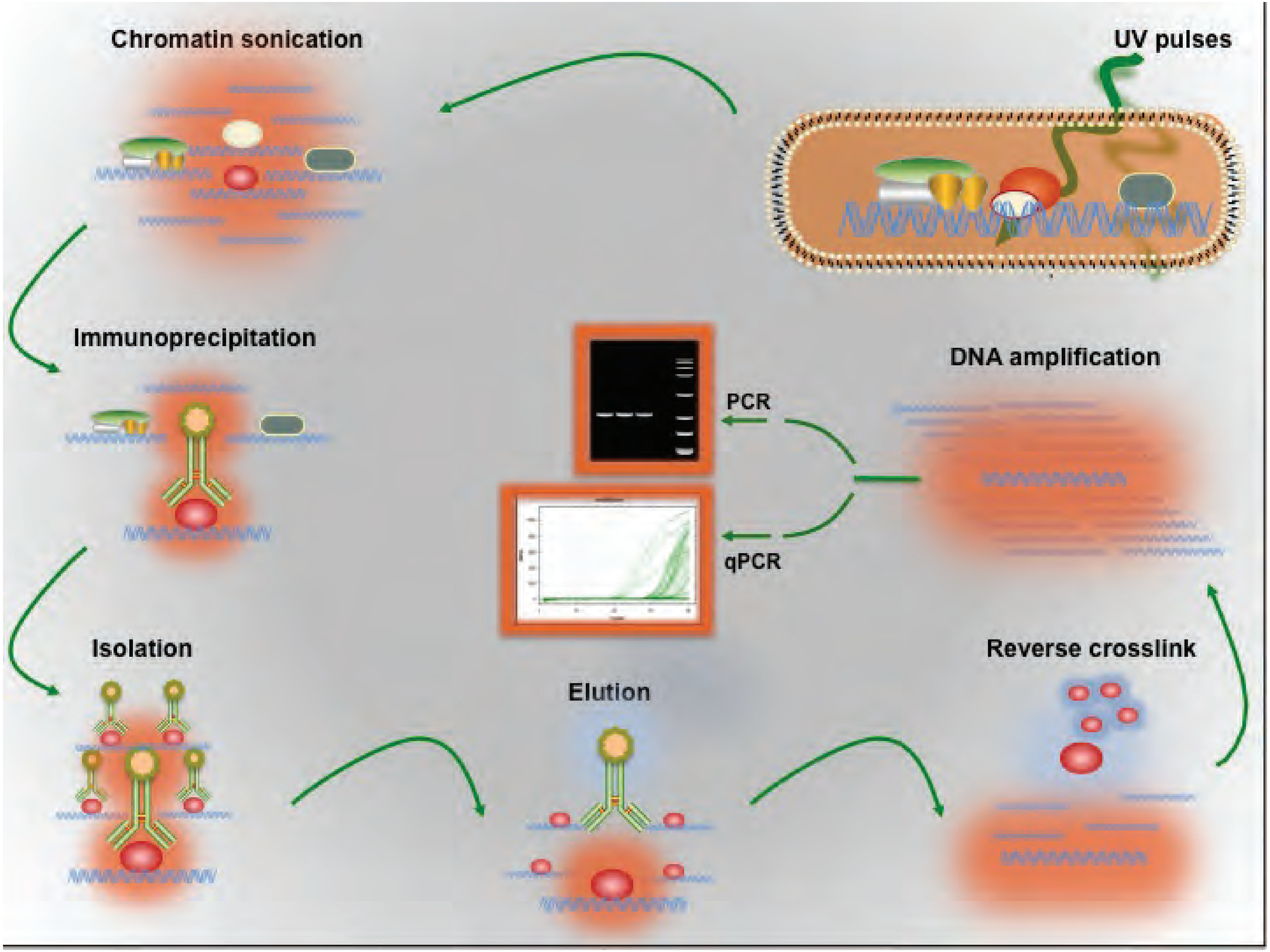


Supplementary Figure S2: Chromatin immunoprecipitation scheme.

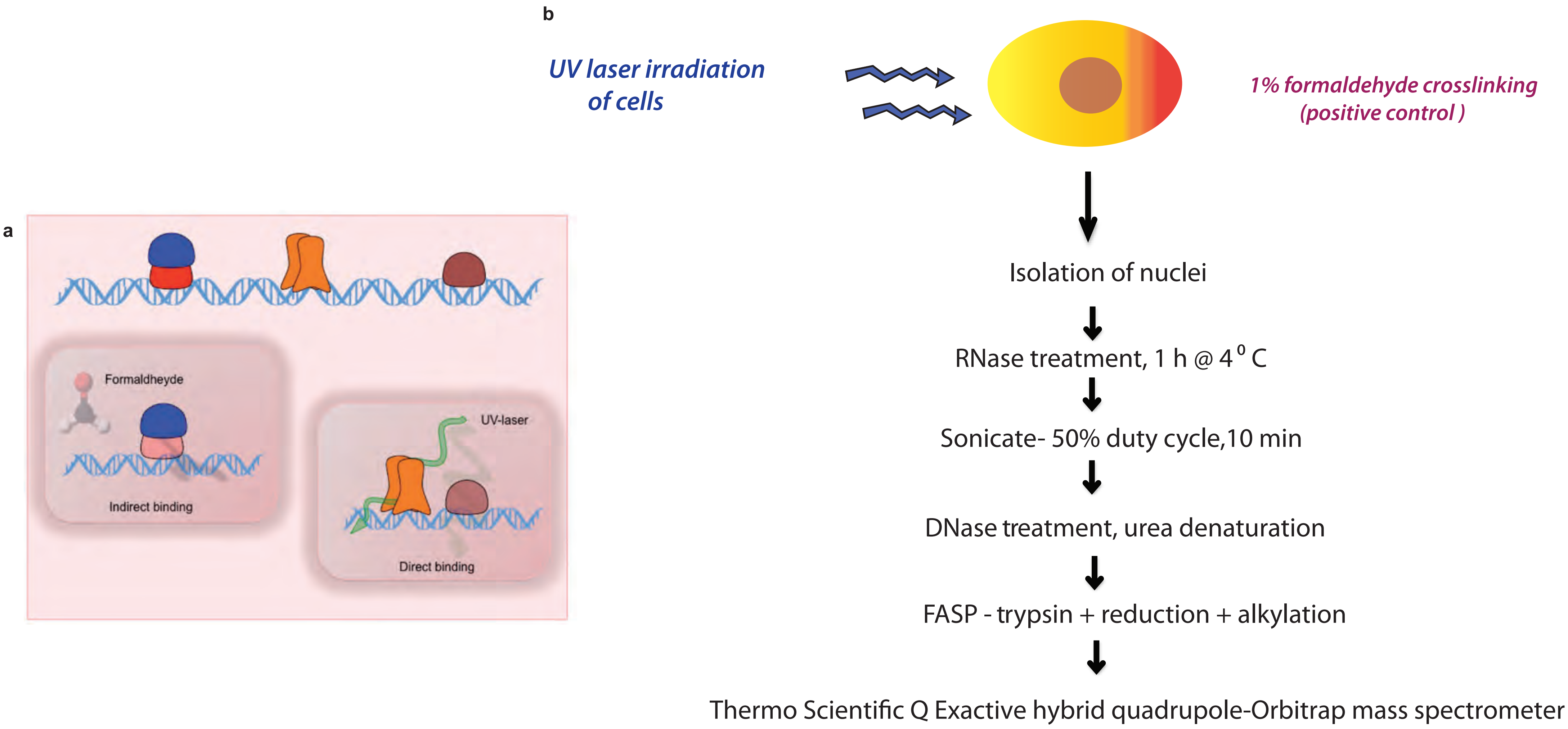


Supplementary Figure S3: (a) Formaldehyde- and UV laser-mediated bindings; (b) MS/MS procedure scheme.

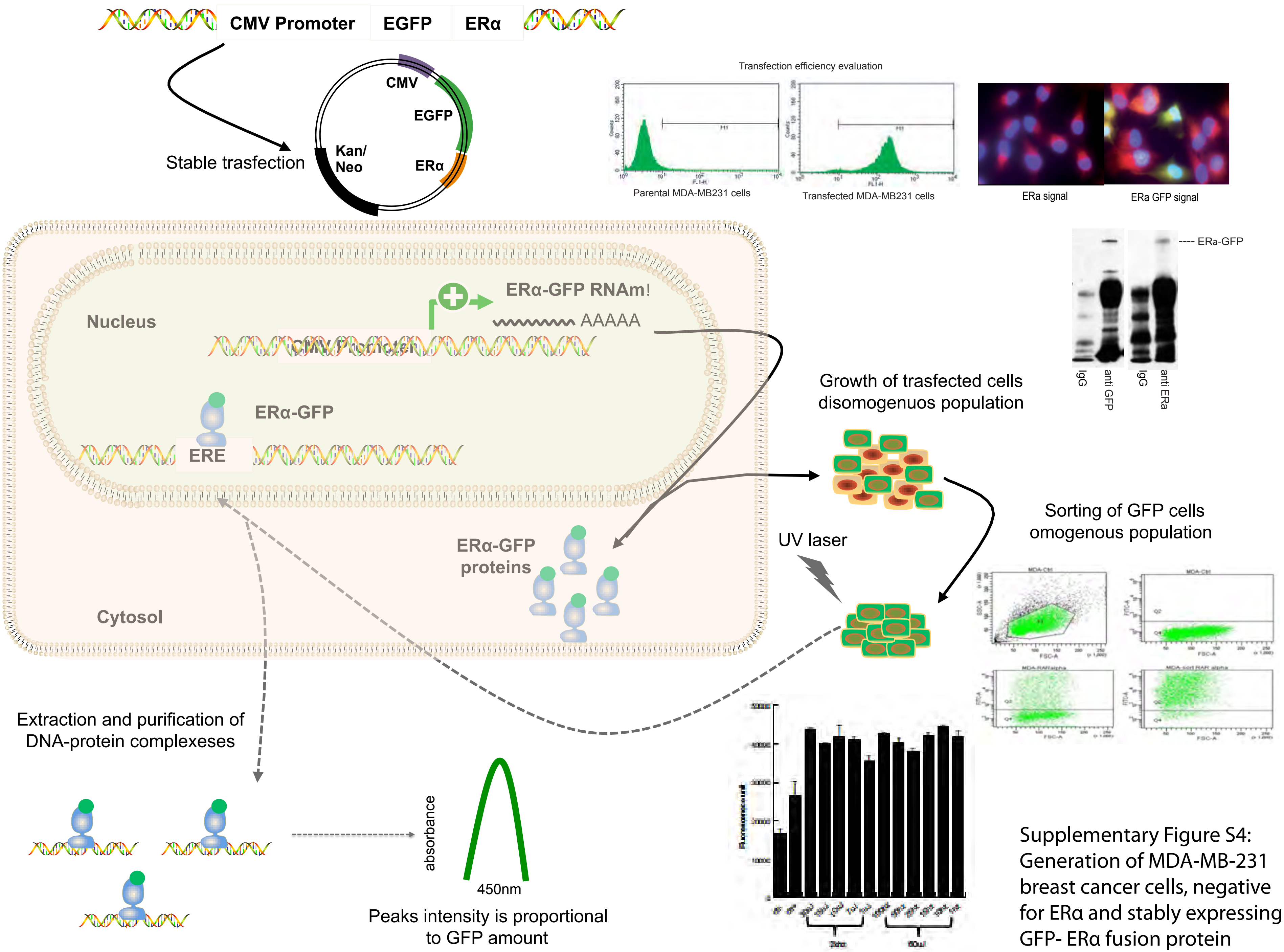


Supplementary Figure S4: Generation of MDA-MB-231 breast cancer cells, negative for ERα and stably expressing GFP- ERα fusion protein

| RR (Hz) | Pulse energy (µJ) | ChIP | photos and counts | PI | Fluorescence | WB | Comet assay | eosin-ematoxylin coloration | ROS | Caspase | Dimers | Cell cycle | WGA staining | Phalloidin staining | Total energy (Hz*µJ) |
|---|---|---|---|---|---|---|---|---|---|---|---|---|---|---|---|
| 1 | 60 | | | | | | | | | | | ✔ | | | 60 |
| 10 | 60 | | | | | | | | | | | ✔ | | | 600 |
| 6 | 150 | | ✔ | | | | | | | | | ✔ | | | 900 |
| 7,2 | 125 | | ✔ | | | | | | | | | ✔ | | | 900 |
| 9 | 100 | | ✔ | | | | | | | | | ✔ | | | 900 |
| 15 | 60 | | ✔ | | | | | | | | | ✔ | | | 900 |
| 60 | 15 | | | | | | | | ✔ | | | ✔ | | | 900 |
| 10 | 140 | | | | ✔ | | | | | | | | | | 1400 |
| 25 | 60 | | | | | | | | | | | ✔ | | | 1500 |
| 15 | 125 | | | | ✔ | | | | | | | | | | 1875 |
| 2000 | 1 | | | | | | | | | | | ✔ | | | 2000 |
| 50 | 60 | | | | | | | | | | | ✔ | | | 3000 |
| 30 | 125 | | | ✔ | ✔ | | | | | | | ✔ | | | 3750 |
| 30 | 140 | | | | | | | | | | | | | | 4200 |
| 50 | 100 | | | ✔ | | | | ✔ | | | | | | | 5000 |
| 48 | 125 | | ✔ | | | | | | | | | ✔ | | | 6000 |
| 40 | 150 | | ✔ | | | | | | | | | ✔ | | | 6000 |
| 60 | 100 | | ✔ | | | | | | | | | ✔ | | | 6000 |
| 100 | 60 | | ✔ | | | | | | | | | ✔ | | | 6000 |
| 60 | 125 | | | ✔ | ✔ | | | | | | | ✔ | | | 7500 |
| 1000 | 8 | | | | ✔ | | | | | | | | | | 8000 |
| 100 | 110 | | | | ✔ | | | | | | | | | | 11000 |
| 200 | 60 | | | ✔ | | | | | | | | | | | 12000 |
| 100 | 125 | | | ✔ | ✔ | | | | | | | ✔ | | | 12500 |
| 100 | 140 | | | | ✔ | | | | | | | | | | 14000 |
| **2000** | **7** | ✔ | ✔ | ✔ | ✔ | | ✔ | ✔ | ✔ | ✔ | ✔ | ✔ | ✔ | ✔ | 14000 |
| 2000 | 8 | | | | | | | | | | | ✔ | | | 16000 |
| 2000 | 10 | | ✔ | | | | | | | | | ✔ | | | 20000 |
| 200 | 100 | | ✔ | | | | | | | | | ✔ | | | 20000 |
| 160 | 125 | | ✔ | | | | | | | | | | | | 20000 |
| 134 | 150 | | ✔ | | | | | | | | | ✔ | | | 20100 |
| 200 | 125 | | | | ✔ | ✔ | ✔ | ✔ | | | ✔ | | | | 25000 |
| 2000 | 15 | | | ✔ | ✔ | ✔ | ✔ | ✔ | ✔ | ✔ | ✔ | ✔ | | | 30000 |
| 20000 | 2 | | | | ✔ | | | | | | | | | | 40000 |
| 5000 | 8 | | | | ✔ | | | | | | | | | | 40000 |
| 7000 | 7 | | | | | ✔ | | | ✔ | | ✔ | | | | 49000 |
| 400 | 125 | | | ✔ | ✔ | | | | | | | ✔ | | | 50000 |
| 2000 | 30 | | ✔ | ✔ | ✔ | ✔ | ✔ | ✔ | ✔ | ✔ | ✔ | ✔ | | | 60000 |
| 480 | 125 | | | ✔ | ✔ | ✔ | ✔ | ✔ | | | ✔ | ✔ | | | 60000 |
| 400 | 150 | | ✔ | | ✔ | ✔ | ✔ | ✔ | | | ✔ | ✔ | | | 60000 |
| 600 | 100 | | ✔ | | ✔ | ✔ | ✔ | ✔ | | | ✔ | ✔ | | | 60000 |
| 500 | 125 | | | | ✔ | | | | | | | | | | 62500 |
| 480 | 150 | | ✔ | | | | | | | | | | | | 72000 |
| 2000 | 60 | | | ✔ | ✔ | ✔ | ✔ | ✔ | ✔ | ✔ | ✔ | ✔ | | | 120000 |
| 1000 | 125 | | | ✔ | ✔ | | | | | | | ✔ | | | 125000 |
| 20000 | 8 | ✔ | | ✔ | ✔ | ✔ | ✔ | ✔ | | | ✔ | | | | 160000 |
| 2000 | 90 | | | | ✔ | ✔ | ✔ | ✔ | ✔ | ✔ | ✔ | ✔ | | | 180000 |
| 2000 | 125 | ✔ | | ✔ | ✔ | ✔ | ✔ | ✔ | ✔ | ✔ | ✔ | ✔ | ✔ | ✔ | 250000 |
| 2000 | 140 | | | | ✔ | | | | | | | | | | 280000 |
| 20000 | 14 | | | | ✔ | | | | | | | | | | 280000 |
| 2000 | 150 | | | | ✔ | | | | | | | | | | 300000 |

- ChIP
- photos and counts
- PI
- Fluorescence
- WB
- Comet assay
- eosin-ematoxylin coloration
- ROS
- Caspase
- Dimers
- Cell cycle
- WGA staining
- Phalloidin staining

**Supplementary Table S1**: Different irradiation conditions used.

**Supplementary Table S2**: Different irradiation conditions used.

| RR (Hz) | Pulse energy (µJ) | | | | | | | | | | | | | | 260nm | 300nm | Total energy (Hz*µJ) |
|---|---|---|---|---|---|---|---|---|---|---|---|---|---|---|---|---|---|
| 2000 | 1 | | ✔ | | | | | | | | | | | | ✔ | ✔ | 2000 |
| 2000 | 3 | | ✔ | | | | | | | | | | | | ✔ | ✔ | 6000 |
| 2000 | 1 | | ✔ | | | | | | | | | | | | ✔ | ✔ | 8000 |
| **2000** | 1 | ✔ | ✔ | ✔ | ✔ | ✔ | ✔ | ✔ | | | ✔ | | | | ✔ | ✔ | 14000 |
| 7000 | 7 | | ✔ | | | ✔ | | | | | | | | | ✔ | ✔ | 49000 |

- ChIP
- photos and counts
- PI
- Fluorescence
- WB
- Comet assay
- eosin-ematoxylin coloration
- ROS
- Caspase
- Dimers
- Cell cycle
- WGA staining
- Phalloidin staining
